# Depression as a Major Component of Public Health for Older Adults

**Published:** 2007-12-15

**Authors:** Daniel P Chapman, Geraldine S Perry

**Affiliations:** National Center for Chronic Disease Prevention and Health Promotion, Centers for Disease Control and Prevention; National Center for Chronic Disease Prevention and Health Promotion, Centers for Disease Control and Prevention, Atlanta, Georgia

## Abstract

Although public health is often conceptualized only as the prevention of physical illness, recent data suggest that mental illnesses are increasingly relevant to the mission of disease prevention and health promotion. Projections are that by 2020, depression will be second only to heart disease in its contribution to the global burden of disease as measured by disability-adjusted life years. Also, as the population ages, successive cohorts of older adults will account for increasingly larger segments of the U.S. population. We present the diagnostic criteria for, prevalence of, and risk factors for depressive disorders among older adults; the challenges of recognizing and treating depression in this population; the cost-effectiveness of relevant public health interventions; and the public health implications of these disorders.

## Introduction

Depressive disorders, which are syndromes characterized by the impairment of mood regulation, most commonly include major depression and dysthymia, a disorder characterized by chronic low mood (1,2). In older adults (generally defined as aged 65 years or older), these disorders may also be characterized by impairment in cognition, a syndrome sometimes referred to as *pseudodementia* (2), and by psychomotor agitation or retardation (1). As a result, symptoms of depressive disorder are frequently masked in older adults and may initially appear to be cognitive impairment or an early sign of neuroendocrine and related chronic disorders, making physical and laboratory examination of older adults with symptoms of depressive disorders important to their care.

Although research shows that the prevalence of major depression is generally lower among older adults than among young adults (3), understanding depressive disorders among older adults remains vital to public health. Rates of major depression rose markedly over the past decade (4), suggesting that future cohorts of older adults will have increasing numbers of people who have experienced or are contending with depressive disorders. Projections suggest that by 2020, depression will become the second leading cause of disease worldwide, as measured by disability-adjusted life years (5). Furthermore, depression characteristically complicates the course and outcome of other illnesses among older adults*.*

Perhaps more compelling, depressive disorders are strong predictors of suicide for older adults, and the majority of those who committed suicide had seen their health care providers within the month before their deaths (6). Despite the lower rates of depression among older people (3), suicide rates are higher in this age group than in any other (7), suggesting that significant depressive symptoms may indicate a serious threat to the health and survival of older adults*.*

Tragically, both older adults and their health care providers may be misguided by the belief that depression is an expected part of aging (8). Also, depressed older adults may have multiple complaints (9), making the diagnosis and treatment of depressive disorders particularly difficult (8,9). These findings suggest the need for public health interventions to destigmatize the diagnosis and treatment of depression and to better enable older adults and their health care providers to recognize this condition. To help better address these issues, we describe recent developments in understanding the characteristics of depressive disorders in older adults and provide an overview of the diagnostic criteria, prevalence, risk factors, and public health impact of these disorders.

## Methods

We searched PubMed (National Center for Biotechnology Information, Bethesda, Maryland) using the keywords *depression* and *dysthymia* crossed with the search terms *public health* and *older adults* and found 51 articles relevant to our study. We limited our review to articles that were published in the past 10 years and that provided definitional or diagnostic criteria for depressive disorders, indicated a specified observation interval, and for the most part, reported on empirical investigations. Subject matter experts suggested additional articles. Table 1 describes the 19 articles retained for comprehensive review.

## Diagnostic Criteria for Depression and Dysthymia

According to the *Diagnostic and Statistical Manual of Mental Disorders*, Fourth Edition (1), the diagnosis of major depression can be made if a patient has five or more of the following symptoms during the same 2-week interval with at least one of the symptoms being either depressed mood or loss of interest or pleasure in activities that were previously pleasurable:

- Depressed mood
- Loss of interest or pleasure in previously pleasurable activities
- Significant weight gain or loss
- Insomnia or hypersomnia
- Psychomotor agitation or retardation
- Fatigue
- Feelings of worthlessness or inappropriate guilt
- Impaired concentration
- Recurrent thoughts of death

Although some empirical investigations use these criteria to determine eligibility for inclusion as study subjects, these investigations must be distinguished from studies using clinically significant depressive symptoms, because the latter may include some study subjects who meet diagnostic criteria for major depression. For these study subjects, the existing symptoms are generally viewed as significantly impairing quality of life and performance of the activities of daily living.

Although similar to a diagnosis of depression, a diagnosis of dysthymia requires only two or more of the following symptoms:

- Poor appetite or overeating
- Insomnia or hypersomnia
- Fatigue
- Low self-esteem
- Impaired concentration
- Feelings of hopelessness

A diagnosis of dysthymia also requires that the person experience depressed mood for most of the day, more days than not, across an interval of at least 2 years, and not be asymptomatic for longer than 2 months during the 2-year course of illness (1). Thus, dysthymia follows a more chronic course than that of depression but comprises fewer disabling symptoms.

Although generally construed as less severe than major depression, dysthymia is by no means a benign illness. In an investigation of older outpatients with a diagnoses of "double depression" (i.e., major depression combined with dysthymia), Joiner et al found that these people had sharply higher rates of hopelessness than did people with a diagnosis of just one of these illnesses (29).

## Prevalence, Comorbidity, and Risk Factors

Major depression has been found to be less prevalent among older adults living in communities than among younger community residents. This finding may appear counterintuitive to notions of depression as an expected facet of aging and may reflect, at least among women, the influence of cohort effects (10). Using the diagnostic criteria for major depression, the Epidemiologic Catchment Area (ECA) Study reported a 1-year prevalence of this disorder of 0.9% among people aged 65 years or older, compared with 2.3% for people aged 45 to 64 years and 3.9% for people aged 30 to 44 years (3). Although the ECA Study began in 1980, its results on major depression remain noteworthy because, unlike other studies, the findings of the ECA Study are based on diagnostic criteria for depressive disorder. Moreover, the ECA Study has the strength of having assessed a large sample of adults aged 65 years or older (N = 187,161) in five geographically distinct sites (New Haven, Connecticut; Baltimore, Maryland; St. Louis, Missouri; Durham, North Carolina; Los Angeles, California) (11).

The Established Populations for Epidemiologic Studies of the Elderly, a longitudinal study of data from three communities (East Boston, Massachusetts; New Haven, Connecticut; and two counties in Iowa), compared over a 6-year interval 5751 older adults who were not depressed with 496 who were depressed. The depressed group had a relative risk of 1.67 (95% CI, 1.44–1.95) for inability to perform activities of daily living and of 1.73 (95% CI, 1.54–1.94) for mobility impairment (12). These results suggest that although depressive disorders may not be highly prevalent among older adults, they pose serious consequences to health and functioning*.*

The living environment of older adults appears highly relevant to the prevalence of depressive disorders. One study of 539 older adults receiving health care in their homes found that about 13.5% were depressed according to diagnostic criteria (13), and a study of the medical records of 3710 nursing home patients found a 20.3% prevalence of depression (14). A study of 562 residents in 65 nursing homes in the Netherlands is particularly telling: symptoms of depressive disorder were pronounced among newly admitted nursing home residents (26.9%), especially among those admitted from their own homes (34.3%) rather than from a hospital (19.7%) (15). Similarly, an epidemiologic study of the prevalence of dysthymia revealed a lifetime prevalence of 1.7% among older adults residing in their home community (3), whereas a study of 224 consecutively diagnosed older outpatients in a late-life depression clinic found a 17.9% prevalence of dysthymia (16).

Given the high rates of depression among older adults receiving home care or living in institutions, it is not surprising that comorbidity in people in these groups may be risk factors for depression. Analyzing data obtained from 2611 Asian adults aged 55 years or older, Niti et al (17) found the prevalence of symptoms of depressive disorder to be much higher for respondents with chronic disease (13.7%–24.2%) than for those without chronic disease (7.5%). Multivariate analyses by these investigators found that after adjustment for comorbidity and functional status, asthma and chronic obstructive pulmonary disease, gastric problems, arthritis, and heart failure remained independently associated with symptoms of depressive disorder. Thus, the chronic diseases frequently reported by older adults may increase the likelihood of depressive disorder.

On the other hand, depressive disorders are themselves associated with risk factors for chronic disease in older adults. In one study, older adults with elevated scores on a test of psychological distress were found to be more likely than their peers not experiencing such distress to smoke, to be obese, to be impoverished, and to have received a diagnosis of diabetes, heart disease, or stroke (18). Older adults who reported a decline in self-reported physical activity over a 3-year interval were significantly more likely to be depressed than were those who did not (19). Likewise, another study found that older adults who were depressed at baseline were less likely than those who were not depressed to report substantial improvement in self-rated health and more likely to report a substantial decline across the 2-year follow-up interval (20). Longitudinal research has established that long-term symptoms of depressive disorder are inversely related to health among older residents of communities (21), corroborating cross-sectional findings that depression is associated with disability in the cognitive and physical activities of daily living (22).

Serebruany et al (30) note that the diagnosis of depression is an independent risk factor for mortality among patients with acute coronary syndromes (ACS). One class of antidepressant medications, serotonin-specific reuptake inhibitors (SSRIs), is thought to inhibit platelet activity, which may protect the heart independent of its use as an antidepressant (30). This property may be particularly valuable because depression, which commonly follows ACS, is associated with an increased risk of mortality (23). In a randomized, double-blind, placebo-controlled study of 369 patients with ACS and depression, Glassman et al (23) found that 53% of depressive episodes began before hospitalization for the index episode of ACS. Over a 4-year study period, the following groups of patients benefited from administration of an SSRI agent: patients with episodes of depression preceding their ACS, patients with a history of depression, and patients whose episodes were severe. The investigators further noted that these three predictors of response to an SSRI medication are independent and specified that the presence of more than one of them considerably increases the benefits of an SSRI but also reduces the probability of spontaneous recovery. These data point to the importance of considering both somatic and psychiatric factors in attempting to optimize the care of older adults with cardiovascular disease, and more broadly, they indicate the interrelatedness of the pathophysiology of organ systems.

## Public Health Impact and Impediments to Intervention

The importance to public health of the finding that depressive disorders may often lead to chronic disease cannot be overstated. We also need to appreciate the tendency of depressive disorders to complicate the course and treatment of chronic disease. Moreover, as chronic disease and depressive disorder are increasingly recognized as contributing to the challenges of providing quality health care, understanding the connection between the two becomes more vital.

Unfortunately, detecting depressive disorders in older adults may be difficult because symptoms may be masked as physical complaints, particularly among frail older adults. Brief assessment tools, such as the Psychological Distress Inventory (PDI-29), may be useful in identifying undiagnosed psychological disorders among frail adults receiving home care services (24) and thereby may decrease the likelihood that depressed older adults will not receive treatment. Other risk factors for nontreatment or inadequate treatment of depression in older adults include being male, being African American or Latino, experiencing fewer than two previous depressive episodes, and expressing a preference for counseling instead of antidepressant medication (25).

Because older adults are usually no longer employed, the cost of depression and the efficacy of its treatment often receive little consideration. Depression in older adults is costly, however, because it results in more visits to doctors' offices and emergency rooms (31). Older adults with chronic disease and depressive disorder may experience increased symptoms of disease (26), and depression is an independent risk factor for mortality (27). Whether alone or with physical chronic diseases, depression is a major source of disability among older adults (32), and older adults with increased symptoms of depressive disorder are less mobile and report fewer social contacts than do their peers who are not depressed (12).

In truth, public health interventions need not be costly and may result in reduced expenditures. The IMPACT (Improving Mood — Promoting Access to Collaborative Training) program, a collaborative-care approach to the management of depression and diabetes in older adults, is a stepped-care program that demonstrates this point (28). Older adults are assigned depression care managers who provide structured activities, including exercise. Participants may choose either problem-solving treatment or antidepressant treatment, both from a primary care provider. The problem-solving treatment is a structured 6- to 8-session psychotherapy intervention with efficacy comparable to that of antidepressants. In the IMPACT Study (recruitment, 1999–2001), 418 of 1801 patients at 18 primary care clinics from 8 health care organizations in 5 unspecified states were randomly assigned to receive either the IMPACT intervention (n = 204) or usual care (n = 214). During a 24-month period, participants in the IMPACT program had a mean of 115 more depression-free days than did participants receiving usual care (28).

The broad costs of depression in older adults — premature mortality, morbidity, and diminished quality of life — are incalculable. Unfortunately, the stigmatization of mental illness and the cost of medication keep many older people from adhering to treatment for depression (33). By integrating depression and other mental illnesses into research and interventions, the public health community will likely increase recognition of depression and lessen needless suffering. At the same time, we need to work to enhance the prevention and management of depression and to address policy and resource considerations necessary to support these endeavors.

## Figures and Tables

**Table.** Studies of Depressive Disorders in Older Adults, 1975–2007

First Author, Study Type	Year	Data Source and Sample Size	Findings
**Prevalence, Comorbidity, and Risk Factors**			
Kasen (10), cohort study	1975, 1983	Mothers in two counties participating in a study of childhood behaviors, New York N = 701	Revealed a cohort effect on the relationship between age and depression, with depression decreasing with age in the cohort born after 1944 (β = −0.26, *P* = .03), compared with the cohort born before 1944 (β = .09, *P* = .18).
Leaf (11), multi-site cross-sectional study	1980	Epidemiologic Catchment Area Study, United States N = 187,161	Prevalence of major depression was lower among adults aged 65 years or older than among adults aged 30-44 years and 45-64 years (3).
Penninx (12), multisite longitudinal survey	1982-1983 (baseline)	Established Populations for Epidemiologic Studies of the Elderly, United States N = 6247	Over a 6-year follow-up period, older adults who were depressed at baseline were more likely than those who were not to develop an incident disability in daily living activities (36.1% vs 23.9%, *P* < 0.001) and in mobility (67.1% vs 48.3%, *P* < 0.001).
Bruce (13), cross-sectional study	1997-1999	Visiting Nurses Service of Westchester County, New York N = 539	Among patients receiving in-home care, 13.5% had major depression, which was significantly associated with morbidity, a past history of depression, and reported pain.
Jones (14), cross-sectional study	1996	Medical Expenditure Panel Survey, Nursing Home Component, United States N = 3710	20.3% (95% CI, 18.9%-21.7%) of nursing home residents were depressed. Prevalence was highest among white non-Latino, younger residents, women, and residents with marital status other than never married, better cognition, comorbidities such as heart disease or Parkinson disease, or a 1-2 year stay in a nursing home.
Achterberg (15), observational study	1997	65 nursing homes in the Netherlands N = 562	26.9% of newly admitted patients had depressive symptoms, with a higher prevalence among those admitted from their homes (34.3%) than those admitted from a hospital (19.7%) but not among those admitted from a shelter.
Devanand (16), cross-sectional study	1994	Late-life depression clinic, United States N = 224	Prevalence of dysthymia was 17.9% among 224 consecutively diagnosed depressed older patients; mean age of onset, 55.2 years. Dysthymia appeared to be preceded by major life stressors such as divorce (22.5%), bereavement (17.5%), retirement (12.5%), family problems (10%), financial problems (7.5%), and major medical illnesses (5.0%).
Niti (17), prospective cohort study	2007 (publication)	Singapore Longitudinal Ageing Study, China N = 2611	Older adults without chronic illnesses were less likely to have depressive symptoms (7.5%) than were those with various chronic medical conditions (stroke, 24.2%; gastric problems, 23.7%; heart failure, 22.3%; asthma and chronic obstructive pulmonary disease, 22.3%; osteoporosis, 15.8%; and hypertension, 13.7%), which were independently associated with depressive disorders even after adjusting for comorbidity and functional status.
Pratt (18), cross-sectional survey	2001-2004	National Health Interview Survey, Family Core and Sample Adults component, United States N = 123,610	The prevalence of serious psychological distress (SPD) was lower for adults aged 65 years or older (2.3%-2.5%) than for other age groups (2.6%-4.0%). People with SPD were significantly more likely than people without SPD to smoke, to be obese, to be impoverished, and to have received a diagnosis of diabetes, heart disease, or stroke.
Penninx (19), prospective cohort study	1992-1993 (baseline);1995-1996 (follow-up)	Longitudinal Aging Study, Amsterdam N = 2121	Over the 3-year study period, decline in self-reported physical ability was significantly greater for participants who were depressed at baseline than for those who were not (*P* < .001).
Han (20), prospective cohort study	1993 (baseline);1995 (follow-up)	Assets and Health Dynamics Among Oldest-Old National Survey, United States N = 6714	Older adults who were depressed at baseline were less likely than those who were not depressed to report substantial improvement in self-rated health and more likely to report a substantial decline across the 2-year follow-up interval.
Meeks (21), prospective cohort study	2000 (publication); conducted in 5 waves across 6- month intervals	Probability sample of community residents aged 55 years or older, Kentucky N = 1479	Chronic depressive symptoms were a strong predictor of decline in daily functioning and of having more health problems at follow-up.
Patrick (22), cross-sectional study	1998-1999	Community-based rural Medicaid long-term care service recipients, West Virginia N = 221	Depression was predictive of disability in cognitive and physical activities of daily living, which in turn were predictive of disability in basic activities of daily living. The number of chronic conditions were significantly correlated with depression.
Glassman (23), multicenter randomized control trial	1997-2001	Sertraline Antidepressant Heart Attack Randomized Trial, United States N = 369	53% of patients had an episode of major depressive disorder before hospitalization for the index episode of acute coronary syndrome, with the majority (94%) of the disorders occurring 30 days before hospitalization.
**Public Health Impact**			
Preville (24), cross-sectional study	Data obtained from older adults registering in two community services centers during 1/15/1997-3/31/1998	Study of Frail Elderly Receiving Home Care Services, United States N = 177	Psychological Distress Inventory (PDI-29) was found superior to the Primary Care Evaluation of Mental Disorders (PRIME-MD) in detecting depression and anxiety in older patients who were not cognitively impaired but who reported a stressful life event during the previous 6 weeks.
Unützer (25), cross-sectional study	1999-2001	Analysis of baseline data from Improving Mood — Promoting Access to Collaborative Trial (IMPACT), United States N = 1801	The groups most likely to report nontreatment or inadequate treatment for depression were males, African Americans, Latinos, people who had fewer than two prior depressive episodes, and people with a preference for counseling instead of antidepressant medication.
Ruo (26), cross-sectional study	9/2000-12/2002	Patient Health Questionnaire and assessment of cardiac function parameters (Heart and Soul Study), United States N = 1024	Patients with coronary artery disease and depressive symptoms reported more significant impairments in physical activity, quality of life, and overall health than did patients with coronary artery disease without depressive symptoms.
Schulz (27), longitudinal study	1989-1995	Assessment of association between baseline depressive symptoms and 6-year mortality among men and women in four counties (Cardiovascular Health Study), United States N = 5201 n = 984 decedents	18.9% of baseline participants died within 6 years. Mortality rate was positively associated with strong baseline depressive symptoms. Even when controlling for other relevant predictors, increased depressive symptoms remained a strong, independent predictor of mortality.
Katon (28), randomized controlled trial	7/1999-8/2001 (recruitment); 2006 (publication)	Subgroup analysis of patients with diabetes from IMPACT, United States N = 418	Patients receiving the IMPACT intervention (structured exercise and problem-solving treatment or antidepressant medication) had a mean of 115 more depression-free days than did participants receiving usual care.
